# Aberrant Expression of JAM2 Inhibits Invasion and Migration in Lung Adenocarcinoma

**DOI:** 10.1002/cnr2.70038

**Published:** 2025-01-21

**Authors:** Jun Chen, Yuan Cui, Zhimeng Chen, Hao Ding, Chang Li, Sheng Ju, Cheng Ding, Chun Xu, Jun Zhao, Xin Tong

**Affiliations:** ^1^ Department of Thoracic Surgery The First Affiliated Hospital of Soochow University Suzhou China; ^2^ Institute of Thoracic Surgery The First Affiliated Hospital of Soochow University Suzhou China

**Keywords:** hypermethylation, immune cells infiltration, invasion, JAM2, lung adenocarcinoma, migration

## Abstract

**Background:**

Lung adenocarcinoma (LUAD) is the most common histological subtype of lung cancer. JAM2, a member of the Junctional adhesion molecule (JAM) family, plays diverse roles in cell–cell contacts and tumor development. Although JAM2's expression and functions have been reported in various cancers, its clinical and biological significance in LUAD remains unclear.

**Aims:**

The aim of this study was to investigate the expression and function of JAM2 in LUAD, and to assess its potential as a prognostic gene and a molecular target for early diagnosis and targeted therapy.

**Materials:**

Immunohistochemistry (IHC) was performed on 37 pairs of LUAD tissues. Gene Ontology (GO) and Kyoto Encyclopedia of Genes and Genomes (KEGG) analyses were conducted among co‐expression genes in different JAM2 subgroups. In vitro experiments were also conducted to study the migratory and invasive capabilities of LUAD cells when JAM2 was overexpressed.

**Results:**

The study confirmed that JAM2 was downregulated in LUAD, possibly due to methylation. JAM2 emerged as an independent prognostic gene for predicting the outcomes of patients with LUAD. IHC analysis revealed the significance of JAM2 with clinicopathological parameters in LUAD. GO and KEGG analyses provided insights into the biological processes and pathways associated with JAM2. In vitro experiments showed that overexpressing JAM2 significantly suppressed the migratory and invasive capabilities of LUAD cells. Additionally, JAM2 played a crucial role in LUAD inflammatory infiltration, and higher JAM2 expression predicted a better immunotherapy response.

**Conclusion:**

JAM2 may serve as a promising molecular target for early diagnosis and targeted therapy of LUAD. Its downregulation in LUAD, potential role as a prognostic gene, and influence on cell migration, invasion, and inflammatory infiltration make it a valuable target for further research and development of therapeutic strategies.

## 1 Introduction

Lung cancer (LC), one of the most commonly diagnosed oncological diseases, is reported to be the leading cause of cancer‐related deaths worldwide [1]. Nonsmall cell lung cancer (NSCLC) is significantly more common (85%) than small cell lung cancer (SCLC) (15%) and could be further subdivided into adenocarcinoma (LUAD), squamous cell carcinoma (LUSC), and large‐cell carcinoma (LCC) [2]. LUAD is the most prevalent histologic subtype, accounting for 50% of NSCLC cases [3]. During the last decade, owing to the refinements in treatments and a better understanding of tumor biology, LC progress has been transformed remarkably [4]. However, the outcome of patients with LC is still abysmally poor, with the predicted 5‐year survival rate being less than 20% [5]. The prognosis for patients is considered flawed, especially for those with advanced LUAD. This is probably related to the late diagnosis. The early diagnosis rate of LC is only about 15%, and 75% of patients are diagnosed at locally advanced or metastatic stages [6]; therefore, it is crucial to find biomarkers with excellent specificity and sensitivity for early diagnosis and metastasis prediction of LUAD. Moreover, it is beneficial to explore safe and efficient molecular targeted therapy for LC.

Tight junctions (TJs) are the apical junctional complex in epithelial and endothelial cells [7]. They are composed of transmembrane proteins, tight junction‐associated marvel proteins (TAMPs), and cytosolic proteins [8]. The junctional adhesion molecule (JAM) family belongs to the immunoglobulin subfamily and is involved in the regulation of TJ barrier function [9]. There are four members in this family, including JAM‐A, JAM‐B, JAM‐C, and JAM‐like [10]. The JAM family is type I transmembrane glycoproteins, consisting of a short N‐terminal signal peptide, two immunoglobulin‐like domains, one transmembrane domain, and one cytoplasmic tail of variable length containing a C‐terminal PDZ‐domain [11]. JAM‐B, also known as JAM2, is different from other JAM family members. It is expressed in junctions of endothelial cells of different vessels but is mostly localized in high endothelial venules [12, 13]. JAM2 is involved in multiple functions, such as cell–cell contacts, the formation of vascular tubes, the homeostasis of stem cell niches, and the promotion of leukocyte transmigration during inflammation [14, 15, 16]. Different expression profiles and vital roles of JAM2 in tumors have also been studied. Hajjari et al. found that JAM2 was upregulated in gastric adenocarcinoma samples compared with adjacent normal tissues. More importantly, its expression level was higher in high grade tumors than low or intermediate grade tumors [17]. However, in other tumors, the expression of JAM2 was downregulated. It was expressed at lower levels in adenocarcinoma and adenoma than in normal colonic mucosa [18]. Yang Peng et al. also showed that JAM2 had a significantly lower level in breast tumors, and that patients with high JAM2 expression had a good prognosis [9]. Moreover, JAM2 restrains BRCA cell metastasis by inhibiting EMT‐related pathways, improving patients' outcomes [19]. However, the clinical and biological significance of JAM2 in LUAD has not been fully illuminated.

In this study, we aimed to comprehensively explore the expression level, prognostic value of JAM2 in LUAD, and identified its significance as a promising biomarker for LUAD development. In addition, we also evaluated the predictive functions of JAM2 using transcriptome sequences. More importantly, by verifying our findings, the function of JAM2 in LUAD was further explored in LUAD cell lines. In summary, we provide additional data about the function of JAM2 in the tumorigenesis of LUAD.

## 2 Methods and Materials

### 2.1 GEO and TCGA Analysis

The mRNA expression profile of gene chips GSE10072, GSE43458, GSE31210, and GSE32863 were downloaded from the GEO database (https://www.ncbi.nlm.nih.gov/geo/). The RNA sequencing data and corresponding clinical information of LUAD patients were obtained from The Cancer Genome Atlas (TCGA) data portal (https://tcga‐data.nci.nih.gov/tcga/).

### 2.2 IHC Stain and Evaluation

IHC was performed as previously described [20] with some modifications. The antibody used for IHC was purchased from Cloud‐clone Corp (PAB783Hu01, Cloud‐clone Corp, Wuhan). The degree of immunostaining of formalin‐fixed, paraffin‐embedded sections was viewed and scored separately by two independent investigators, who were blinded to the histopathologic features and patient data of the samples. Both the staining intensity and the positive staining rate were divided into four grades (scored from 1 to 4 points), and their product was multiplied to give a final score ranging from 1 to 16. The total expression of JAM2 was determined as either negative or low expression (−), with a score ≤ 6 or high expression (+) with a score > 6.

### 2.3 Human LUAD Tissues

Tumor tissue and normal tissue (taken from a location more than 5 cm from the tumor) from the same patient were considered as pairs. Tumor specimens were obtained from 37 patients with LAC who underwent lung resection without preoperative chemotherapy at the First Affiliated Hospital of Soochow University between 2020 and 2021. After surgical removal, the tissue samples were immediately fixed in formalin and embedded in wax for IHC. The patients were selected randomly. Permission to use the tissue sections for research purposes was obtained from the Ethics Committee of the First Affiliated Hospital of Soochow University and written informed consent was obtained from each patient.

### 2.4 Cell Culture

Human LUAD cell lines A549, H1299 and H1650, immortalized human bronchial epithelial cell lines BEAS‐2B and HBE, as well as human embryonic kidney (HEK) 293T cells were purchased from the Cell Bank of the Chinese Academy of Sciences. These cells were cultured in either RPMI 1640 medium (HyClone, South Logan, UT, USA) or Dulbecco's modified Eagle's medium (DMEM; Thermo Fisher Scientific, Waltham, MA, USA), both supplemented with 10% fetal bovine serum (FBS; Invitrogen, Carlsbad, CA, USA). The cells were maintained in a humidified incubator with 5% CO_2_ at 37°C. The medium was refreshed every 2–3 days.

### 2.5 Western Blot Analysis

Cells were harvested after being washed twice with cold PBS. They were then lysed and collected in RIPA lysis buffer (Beyotime, China), which contained protease inhibitor cocktail (Beyotime, China). The protein concentration was measured using a BCA kit (Beyotime Institute of Biotechnology). The extracted proteins were separated with SDS‐polyacrylamide gel electrophoresis (PAGE) and then transferred to a polyvinylidene fluoride (PVDF) membrane (Millipore, USA). After blocking with 5% skimmed milk for 90 min at room temperature, the membranes were incubated with specific primary antibodies overnight at 4°C. Then, after washing three times with Tris buffered saline containing 0.1% Tween‐20, the membranes were incubated with the secondary antibody for 1 h at room temperature. The protein expression levels were visualized using an ECL detection system (Tanon, China). The primary antibodies employed in Western blot were as follows: Rabbit β‐Tubulin antibody (A17913, ABclonal, China), mouse anti‐JAM2 antibody, mouse anti‐β‐actin antibody, and antirabbit or antimouse secondary antibodies (Santa Cruz Biotechnology, Santa Cruz, CA, USA).

### 2.6 Generation of LUAD Cell Lines Stably Overexpressing JAM2

The JAM2‐HA overexpression vector containing JAM2 coding sequence and an HA tag, was synthesized by Tsingke (Suzhou, China). The empty lentivirus vector was served as a negative control. Subsequently, the JAM2 expression construct or empty vector was co‐transfected with packaging plasmids Helper 1.0 and Helper 2.0 (GeneChem Inc.) into HEK 293 T cells using Lipofectamine 3000 (Invitrogen). At 48 h post‐incubation in DMEM with 10% FBS, the packaged lentiviruses were collected and used to infect A549 and H1299 cells for 72 h. Finally, HA‐tagged JAM2‐overexpressing A549 and H1299 stable cells were selected with 2 μg/mL puromycin (Solarbio Lifesciences, Beijing, China).

### 2.7 Wound‐Healing Assays

The control and treated cells were seeded and cultivated in six‐well plates. After cells grew to 90% confluence, a scratch was generated using a sterilized pipette tip. Then, cells were washed three times with PBS and imaged in six random fields. After cultured in serum‐free medium for 24 h, the cells were washed and imaged. Cell migration distances into the scratched area were determined and analyzed using ImageJ Launcher software (National Institutes of Health, Bethesda, MD, USA).

### 2.8 Cell Migration and Invasion Assays

Transwell assays were performed according to instructions from the manufacturer. For migration assays, 5 × 10^4^ cells were seeded into the upper chamber of the transwell plate in 200 μL RPMI 1640 medium with 1% FBS, and 800 μL complete medium was added to the lower compartment. For invasion assays, the bottom membranes of the transwell chambers were coated with 50 mg/L Matrigel at a 1:8 dilution. Cells were then plated in the same way as in the migration assays. After incubating for 24 h (migration) or 30 h (invasion) in 37°C, the inserts were removed and the nonmigrating or noninvading cells were removed with cotton swabs. Cells that migrated or invaded to the lower chamber were fixed and stained with 1% crystal violet. Finally, cells were photographed and counted in five randomly selected fields.

### 2.9 Bioinformatics Analysis

The DEGs among four GEO data sets were selected by DESeq2 R package. Gene Expression Profiling Interactive Analysis (GEPIA) (http://gepia.cancer‐pku.cn) provides differential RNA expression analysis between LUAD and normal tissues [21]. Methylation status of JAM2 was measured through MEXPRESS, an online tool for the integration and visualization of gene expression, DNA methylation and clinical data from TCGA [22]. To further clarify the relationship between JAM2 expression and LUAD prognosis, we used GEPIA for survival analysis. Gene Ontology (GO) enrichment and Kyoto Encyclopedia of Genes and Genomes (KEGG) pathway analysis of differentially expressed genes (DEGs) were implemented using the clusterProfiler R package. Immune cell infiltration was estimated by ssGSEA R package.

### 2.10 Statistical Analysis

All data were statistically analyzed using R software (version 4.1.2). The Wilcoxon nonparameter test was used for comparisons between two groups, and the Spearman method was used for the correlation analysis. A *p* value < 0.05 was considered statistically significant.

## 3 Result

### 3.1 Identification of JAM2 From GEO Date Sets

**FIGURE 1:**
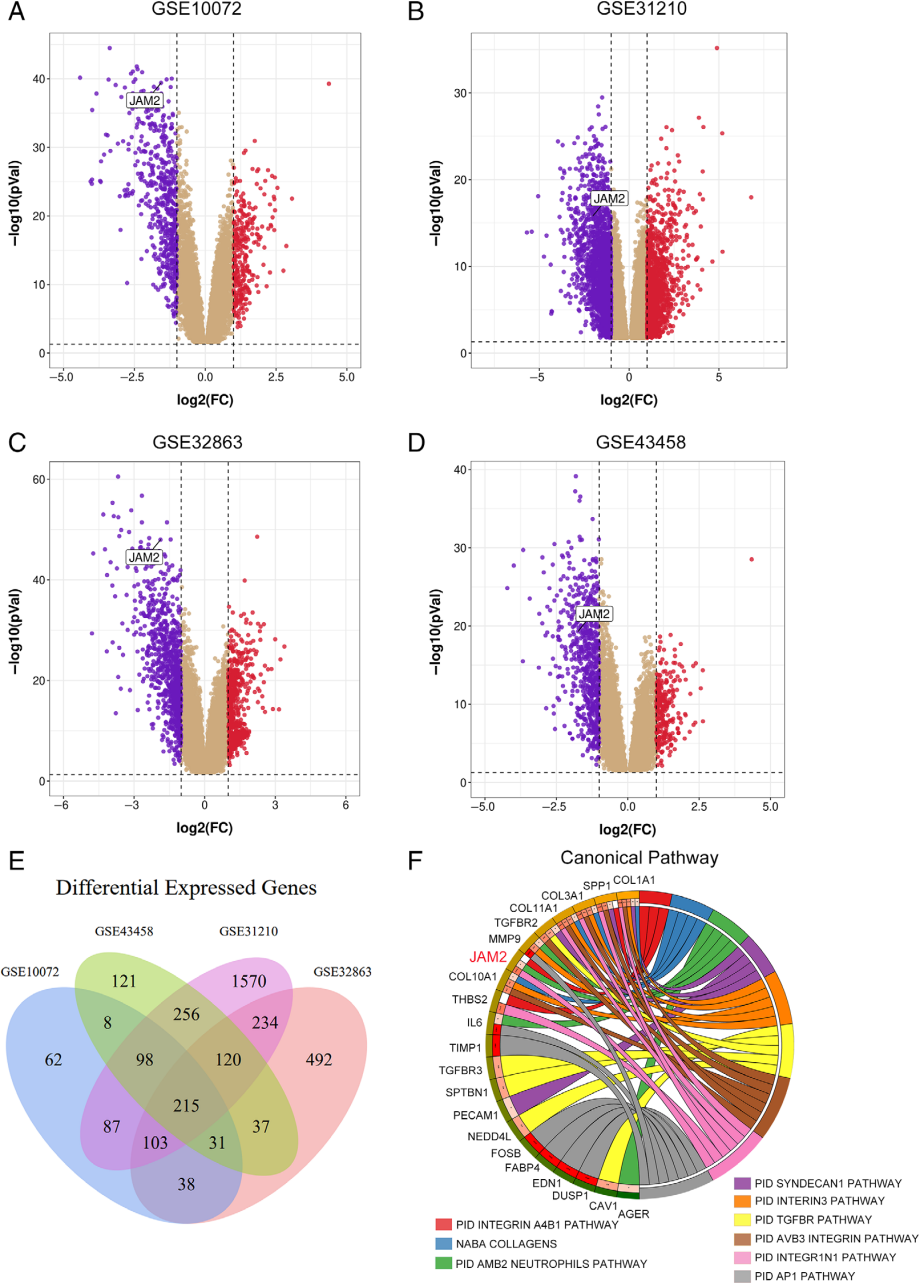
Identification of JAM2 from GEO date sets. (A–D) Volcano plots showing all the expressed genes from GSE10072, GSE43458, GSE31210, and GSE32863, respectively. Blue represents downregulated genes, and red represents upregulated genes. DEGs: differentially expressed gene. (E) Venn diagram for the overlapping DEGs in four date sets. (F) Enrichment analysis of differential genes in signaling pathways related to tumor genesis and development.

GSE10072, GSE43458, GSE31210, and GSE32863 data sets were selected to analyze and identify the DEGs between normal tissues and LUAD. According to the set criteria of *p* value < 0.01 and fold change > 1 or fold change < −1, DEGs of four microarrays were identified. As shown in the volcano plots, there are 846, 945, 3665, and 1334 DEGs, respectively (Figure 1A–D). Then, a Venn diagram was used to overlap among the four datasets, and we found 215 DEGs, among which 59 genes were upregulated, and 156 genes were downregulated (Figure 1E, Table S3). Among them, the top 20 levels of upward and downward gene expression were increased in Table S1. We then conducted enrichment analysis of differential genes and found that genes such as COL1A1, SPP1, COL3A1, TGFBR2, MMP9, and JAM2 were widely enriched in signaling pathways related to tumorigenesis and development (Figure 1F). In this research, we mainly focused on the role of JAM2 in LUAD.

### 3.2 Expression Levels and Prognosis Value of JAM2 in LUAD

**FIGURE 2:**
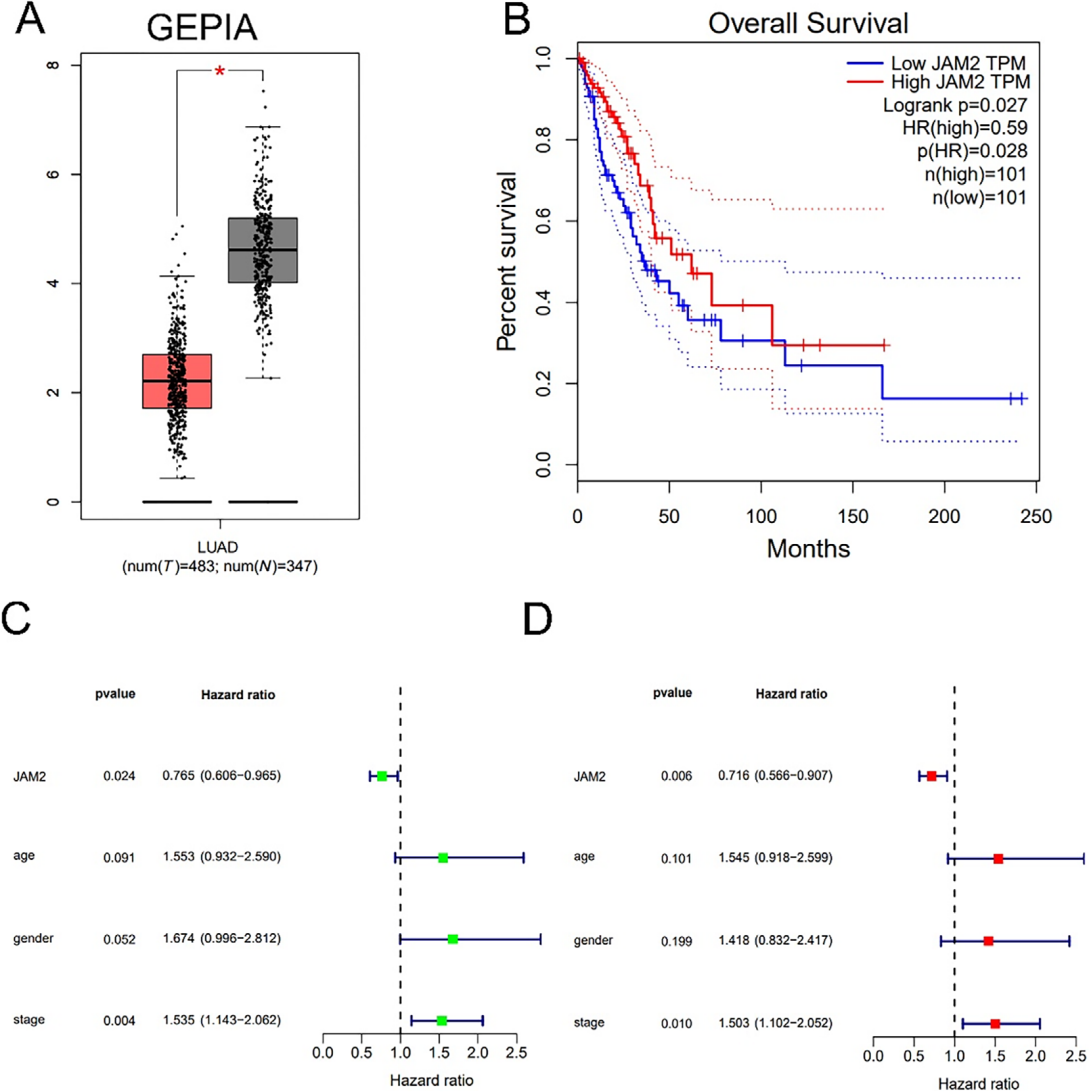
Expression level and prognostic value of JAM2 in LUAD. (A) JAM2 expression levels in LUAD from TCGA data. (B) High expression of JAM2 predicts better prognosis in TCGA cohort. (C, D) Univariate Cox analysis and multivariate Cox analysis showed that JAM2 acts as a protective role in LUAD.

Online database GEPIA was used to explore the expression profiles of JAM2 in LUAD. As we shown in Figure 2A, JAM2 were downregulated in LUAD (*p* < 0.001). Furthermore, to study the association between JAM2 expression level and prognosis, survival association analysis was also performed through GEPIA. The results showed that LUAD patients with higher expression of JAM2 had longer survival times than lower ones (Figure 2B) (*p* = 0.028). More importantly, univariate Cox regression analysis showed that JAM2 expression (hazard ratio = 0.765, *p* = 0.024) and pathological stage (hazard ratio = 1.535, *p* = 0.004) were significantly associated with the prognosis for LUAD (Figure 2C). Multivariate Cox regression analysis confirmed that JAM2 was a protective factor of LUAD (hazard ratio = 0.716, *p* = 0.006). Therefore, JAM2 could be served as an independent prognostic gene to predict the outcomes of patients with LUAD (Figure 2D).

### 3.3 Correlation Between JAM2 Expression and Clinicopathological Parameters in LUAD

**FIGURE 3:**
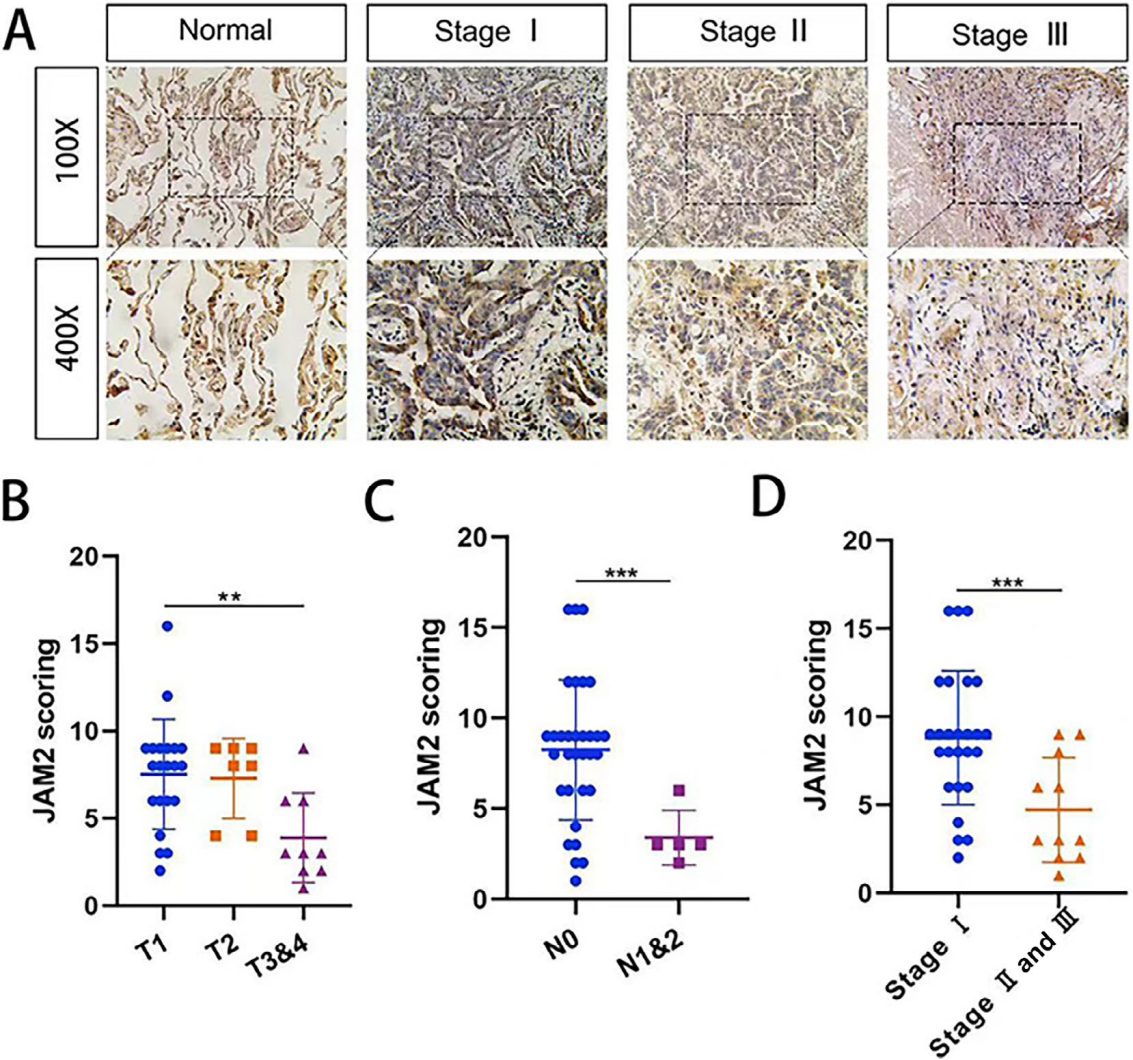
Immunohistochemistry staining of JAM2 in LUAD tissues. (A) Paraffin‐embedded tissue sections were stained with antibodies against JAM2 in normal lung tissues and different stages of LUAD tissues. (B–D) Analysis of JAM2 expression and clinicopathological factors in LUAD, the number of each group was as follows: T1: 21; T2: 7; T3 and 4: 9; N0: 32; N1: 5; Stage I: 26; Stages II and III: 11. *p < 0.05, **p < 0.01, ***p < 0.001.

To gain further insight into the clinical significance of JAM2, IHC for 37 pairs LUAD tissue was performed. Immunostaining of JAM2 was scored on a semiquantitative scale. The staining intensity and the positive staining rate were both divided into four grades (scored from 1 to 4 points), the product of which multiplied to give a final score. As shown in Figure 3A, there was a significant higher expression of JAM2 in normal tissues than tumors. Based on the scores of JAM2, we found that JAM2 expression in patients at stage I was higher than that in patients at Stages II and III (Figure 3A,B). Furthermore, downregulation of JAM2 expression negatively paralleled with the increase of regional lymph node stage and tumor grade (Figure 3C,D). There was no significant correlation between JAM2 expression and other clinicopathological parameters, such as sex or age (*p* > 0.05). These results indicated that JAM2 might be an important biomarker for LUAD progression.

### 3.4 The Expression of JAM2 May Be Involved in Cancer Metastasis

**FIGURE 4:**
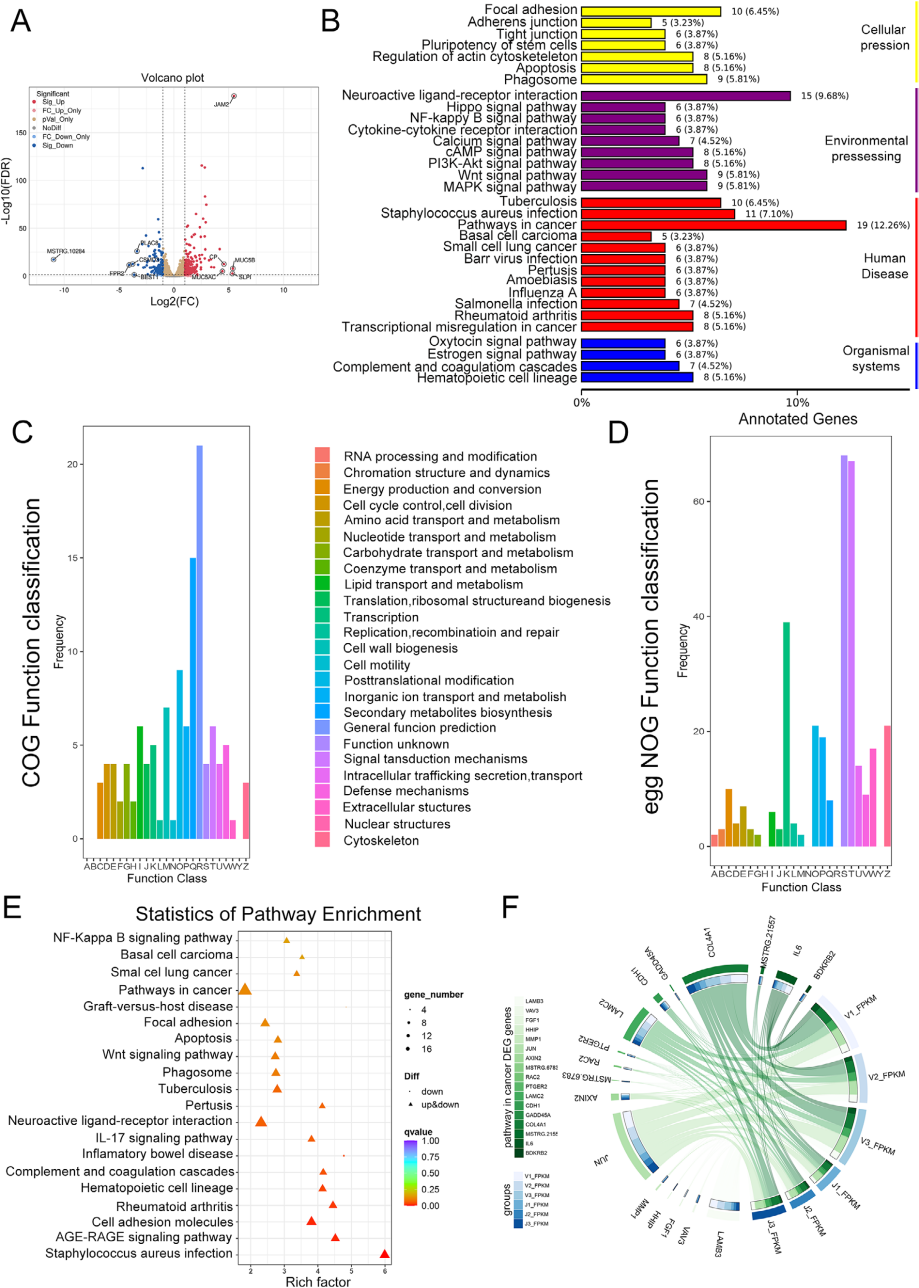
Functional annotation of genes in different JAM2 subtype. (A) Volcano plot of co‐expression genes. (B) Enrichment analysis for the co‐expression genes. (C, D) COG and eggNOG function classification results of co‐expression genes. (E) Pathways analysis of co‐expression genes. (F) Different expressed genes in the cancer pathway.

**FIGURE 5:**
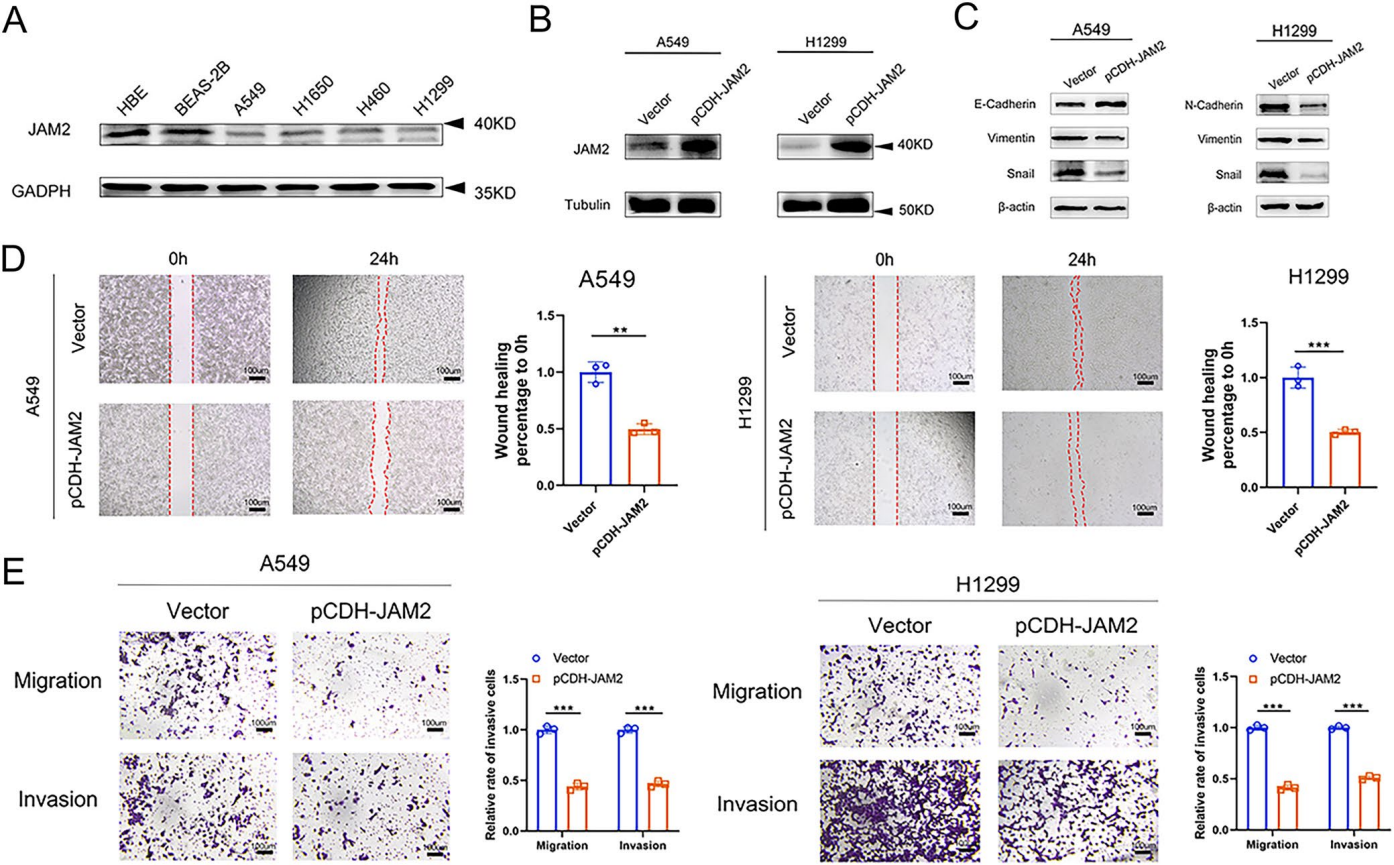
JAM2 downregulated in LUAD cell lines and inhibits the invasion and migration of LUAD. (A) The expression levels of JAM2 among bronchial epithelial cells (HBE, BEAS‐2B) and LUAD cell lines (A549, H1299, H460, and H1650). (B) The efficiencies of JAM2 overexpression in A549 and H1299. (C) The expression of EMT markers was detected by Western blotting. (D) Wound‐healing migration assays were performed on JAM2‐overexpressing A549 and H1299 cells as well as control cells. (E) JAM2‐overexpressing A549 and H1299 cells were allowed to migrate through a polycarbonate membrane or invade through Matrigel‐coated membrane in Transwells. Then, cells were stained, photographed, and counted in at least four random fields under a light microscope. *p < 0.05, **p < 0.01, ***p < 0.001.

Then, we constructed A549 and H1299 cells that stably overexpressed JAM2, and the efficiencies were confirmed by Western blot (Figure 5B). Transcriptome sequencing was performed between vector group and overexpression group in A549, followed by bioinformatic analysis. As shown in Figure 4A, MUC5B, MUC5AC, SLPI, and CP were the most upregulated genes along with overexpression of JAM2, whereas PLAS8, CSMD3, FPR2, and BEST1 were decreased clearly (Figure 4A). The top 10 log2 fold changes of up‐ and downexpression genes have been added in Table S2. Subsequently, enrichment analyses were used to detect the genes that were co‐expressed with JAM2 expression. The common genes were enriched in many cellular processes, such as focal adhesion, TJ, and adherent junction (Figure 4B). We also found that they were relevant to PI3K‐Akt, MAPK, and Wnt signal pathways (Figure 4B). COG function classification revealed a concentration in secondary metabolites biosynthesis, transport, catabolism, inorganic ion transport posttranslational modification, and protein turnover (Figure 4C). Meanwhile, the results of eggNOG focused on signal transduction mechanisms, transcription and cytoskeleton (Figure 4D). The classification of annotated genes showed that the expression of JAM2 was mainly related to focal adhesion, phagosome, and neuroactive ligand–receptor interaction, especially in pathways in cancer (Figure 4E). When we focused on different expressed genes in a pathway related to tumor development, there were some important oncogenes correlated with JAM2, such as JUN, MMP1, CDH1, and so on (Figure 4F). As focal adhesion, TJ, and adherent junction are closely associated with cancer metastasis [23, 24, 25], signal pathways mentioned above might also play crucial roles in LUAD process. These findings support that JAM2 may be involved in cancer development.

### 3.5 JAM2 Is Downregulated in LUAD Cell Lines and Inhibits the Invasion and Migration of LUAD

To further verify the above findings, Western blot was used to investigate JAM2 expression in bronchial epithelial cells and LUAD cell lines. Consistent with the profiles of GEO and TCGA cohorts, JAM2 expression was relatively higher in bronchial epithelial cells (HBE and BEAS‐2B) than in LC cell lines, including A549, H1299, H1650, and H460 (Figure 5A). As mentioned above, we constructed A549 and H1299 cells that stably overexpressed JAM2 (Figure 5B). Subsequently, JAM2 overexpressing in LUAD cells displayed increased E‐cadherin levels, as well as decreased levels of N‐cadherin, snail, and vimentin, suggesting an inhibitory role of JAM2 in EMT (Figure 5C). The blotting images corresponding to the other two independent experiments are shown in Figure S1. Wound‐healing migration assay was employed to observe whether there was an effect on the migration ability of LUAD cell lines after JAM2 overexpression. From the results, we found that the wound recovery rate was significantly reduced after JAM2 overexpression (Figure 5D). In addition, when JAM2 was overexpressed, the migratory and invasive capabilities of A549 and H1299 cells were also significantly suppressed (Figure 5E). So, overexpression of JAM2 can inhibit the invasion and migration of LUAD cells.

### 3.6 Hypermethylation of JAM2 in LUAD

**FIGURE 6:**
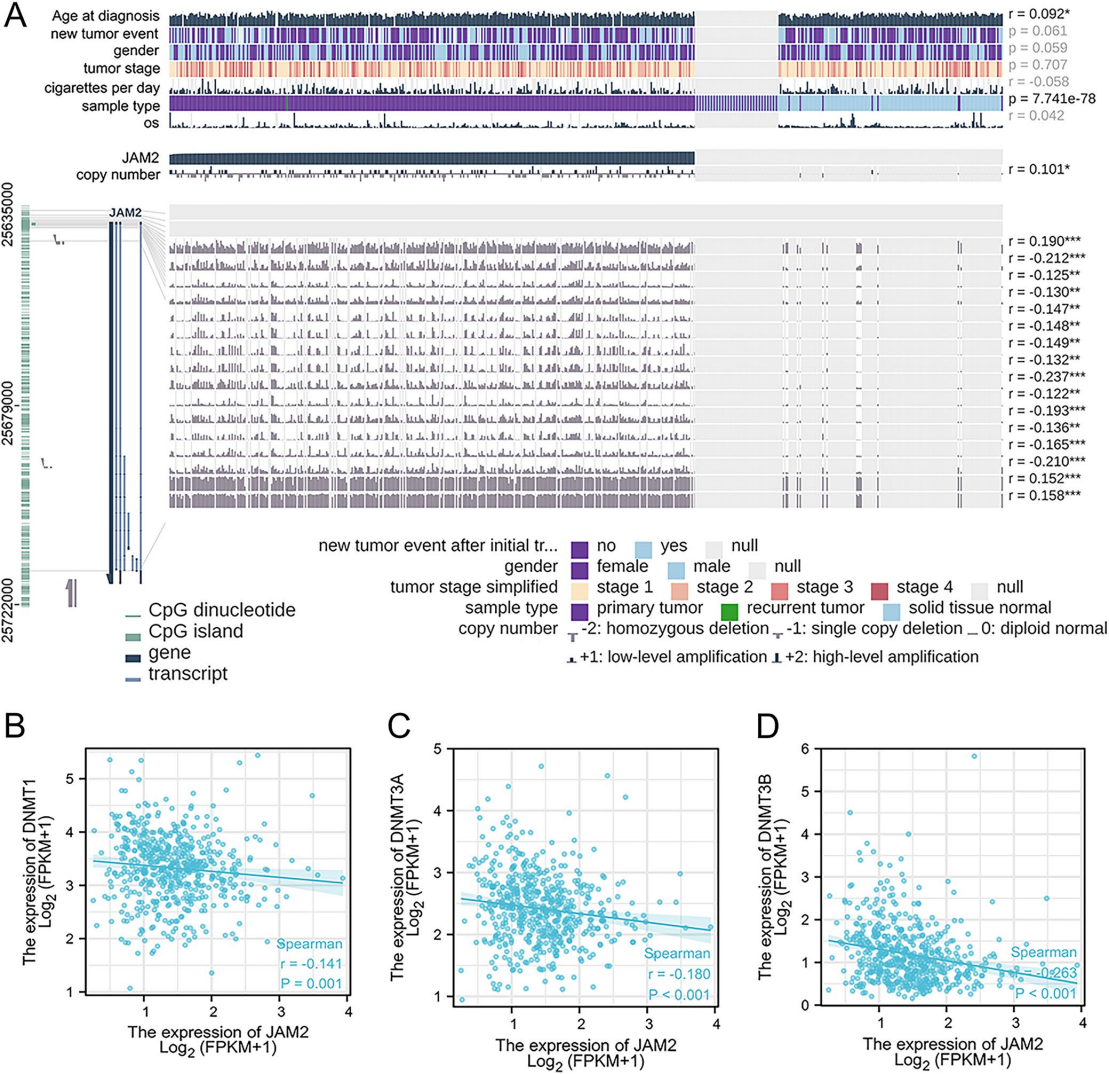
Methylation status of JAM2 in LUAD. (A) JAM2 was significantly more highly methylated in cancer tissues through MEXPRESS. (C, D) JAM2 expression was negatively related to three methyltransferases DNMT1, DNMT3A, and DNMT3B.

It is clear that hypermethylation of the host genome directly targets key tumor suppressor genes and results in gene silencing and inducing tumorigenesis. The methylation status of JAM2 was analyzed to ascertain the downregulated mechanism in LUAD. The results demonstrated that JAM2 was highly methylated in cancer tissues compared to normal tissues (Figure 6A). Spearman correlation analysis of three DNA methyltransferases showed that JAM2 expression was negatively related to the expression of DNMT1 (*r* = −0.14, *p* = 0.001), DNMT3A (*r* = −0.18, *p* < 0.001), and DNMT3B (*r* = −0.263, *p* < 0.01) (Figure 6B–D).

### 3.7 Immune Cells Infiltration Analyses of JAM2

**FIGURE 7:**
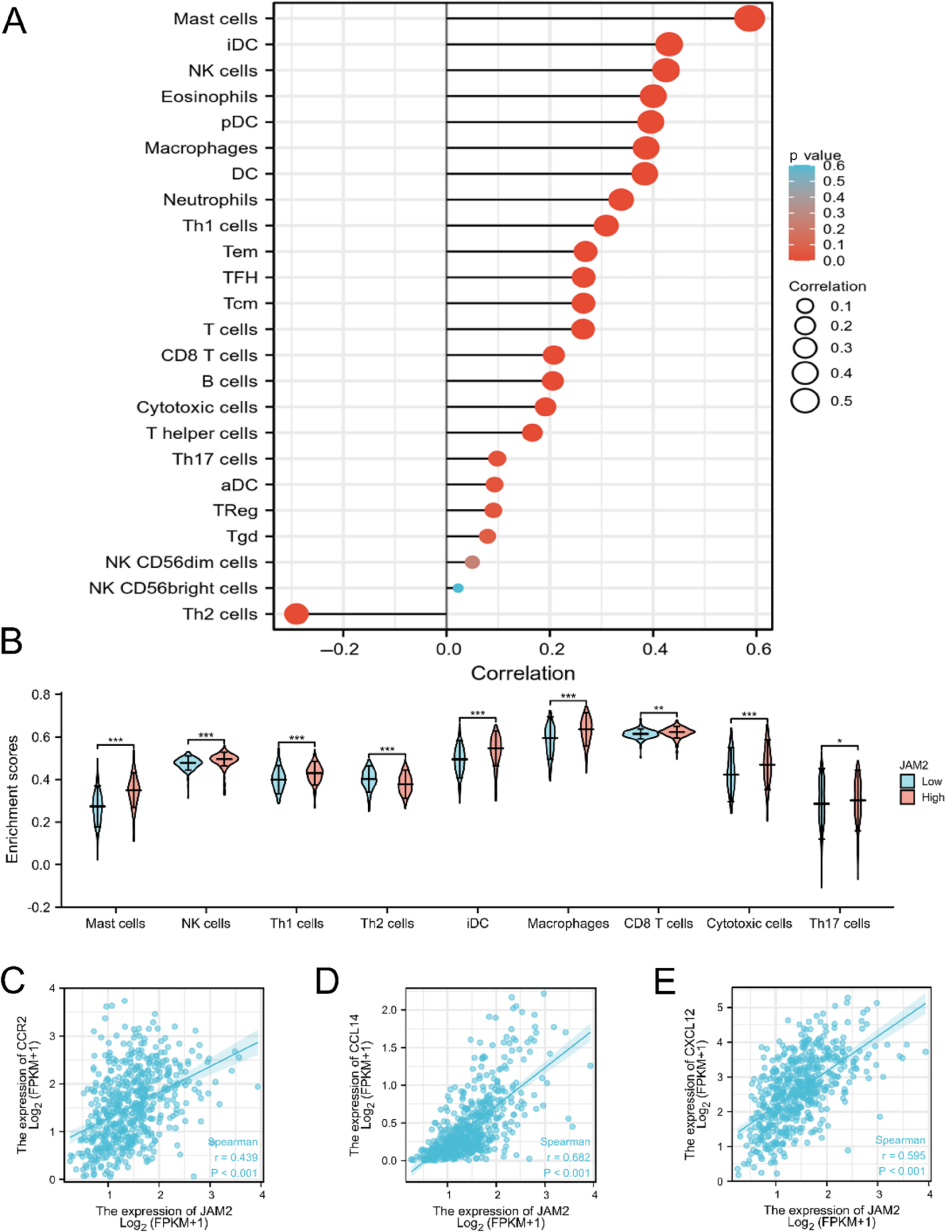
The landscape of the immune infiltration in LUAD. (A) Association of JAM2 expression with the infiltration of 24 immune cell. (B) Violin diagram the proportions of eight immune cells in different JAM2 subgroups. Central dotted line of the violin plot represents median, and upper and lower dotted line represents quartile. Significant statistical differences between the two subgroups were assessed using the Wilcoxon test (ns: not significant, *p < 0.05, **p < 0.01, ***p < 0.001, ****p < 0.0001). (C–E) Correlation analysis of JAM2 and CCR2 (*r* = 0.437; *p* < 0.001), CCL14 (*r* = 0.682; *p* < 0.001), and CXCL12 (*r* = 0.595; *p* < 0.001) in TCGA database.

Immune cells play a vital role in the tumor microenvironment and can affect the prognosis of various cancer. However, it is unclear whether JAM2 has impacts on the recruitment of immune cells. We evaluated the correlation between immune cell infiltration and JAM2 expression by R package ssGSEA. The scores of 24 immune cell types were calculated based on TCGA‐LUAD database. JAM2 expression was positively correlated with immune cells except Th2 cells (Figure 7A). While Mask cells, NK cells, Th1 cells, iDC, Macerphages, CD8^+^T cells, Cytotoxic cells, and Th17 cells were enriched in the JAM2‐high subgroup, Th2 cells were more common in the JAM2‐low subgroup (Figure 7B). It has been reported that chemokines have an inhibitory effect on tumor progression. Therefore, we investigated chemokine levels in different JAM2 subgroups and found a significant positive correlation between JAM2 with CCR2 (*r* = 0.437; *p* < 0.001), CCL14 (*r* = 0.682; *p* < 0.001), and CXCL12 (*r* = 0.595; *p* < 0.001) (Figure 7C–E).

## 4 Disscusion

JAM2 belongs to the immunoglobulin super family (IgSF) of adhesion molecules. It is related to interendothelial cell–cell contacts, the formation of vascular tubes, the homeostasis of stem cell niches, and the promotion of leukocyte adhesion and transmigration [26]. Multiple studies have shown that JAM2 is differently expressed in normal and cancerous tissues. JAM2 was upregulated significantly in tumor samples compared with adjacent normal tissues in gastric cancer [17], glioma, and human oral cancer [27, 28]. On the contrast, it was downregulated in esophageal LUSC, breast cancer, colorectal cancer, and melanoma [18, 29, 30, 31]. In our work, we sought to confirm the expression level of JAM2 in LUAD. As shown above, JAM2 was downregulated in cancer samples than normal lung tissues. We confirmed the results not only in online data sets such as GEO and TCGA, but also in LUAD tissues and cell lines. Interestingly, JAM2 expressed much higher in early stage of tumors, and decreased with the upload of pathological stages. In a word, JAM2 may serve as a biomarker for early stage of LUAD progression.

Metastasis is a major cause of death in patients with LC, during which cancer cells spread to new areas of the body, usually through the blood stream or lymphatic systems. A considerable number of patients with early‐stage LC relapse due to unnoticed distant metastasis [32]. Changes in the cell adhesion ability during cancer progression cause tumor cells to be more dynamic and spread to distant sites. Tumor cells invade various tissues and organs accompanied by the disruption of TJ [25], which was in consistent with our sequence result that overexpression of JAM2 could active the mentioned pathways. In a word, JAM2 leads to the inhibition of tumor invasion.

Numerous studies have shown that JAM2 played an important role in the metastasis of tumor cells. The complex of JAM2 and JAM‐C, which were paracrine stimuli from both tumor cells and endothelial cells, might promote glioma invasion by regulating cell migration and invasion through c‐Src, which is a proto‐oncogene [33]. In addition, overexpression of JAM2 could block the invasion and migration of breast cancer cells, and the mechanism might be that JAM2 inhibits the EMT process [9]. Besides that, JAM2 could directly activate the c‐Src/MMP9 pathway, and JAM2 silencing significantly decreased the migratory and invasive abilities of PanCa cells [10]. JAM‐2 expression levels were negatively correlated with colorectal cancer progression across multiple datasets. This finding suggests that JAM‐2 may play a role as a tumor suppressor in the progression of colorectal cancer. JAM‐2 is involved in biological processes such as intercellular adhesion and signal transduction through interaction with other cell surface molecules. In colorectal cancer cells, reduced expression of JAM‐2 may lead to reduced intercellular adhesion, thereby increasing the migration and invasion ability of tumor cells [34]. Our data ruled out the role for JAM2 in LUAD metastasis. The enrichment results showed that PI3K‐Akt, MAPK, and Wnt signal pathways were significantly changed in the JAM2 overexpression group. Meng et al. found that TRPM7 regulates migration and invasion of metastatic breast cancer cells via MAPK pathway [35]. In gastric cancer, cancer‐associated fibroblasts influences invasion and metastasis through Wnt signaling pathway [36]. Similarly, upregulation of JAM2 inhibited EMT and attenuated the migratory and invasive potential of LUAD cells.

DNA methylation is an imperative epigenetic mechanism that regulates several biological processes. It represents a molecular mechanism associated with gene repression and has been assumed that de novo modification in cancer may contribute to the tumor phenotype. DNA methylation inhibits genes that are initially activated in the source tissue, especially those involved in tumor suppression [37]. We analyzed the methylation status of JAM2 in LUAD. Figure 6A shows significantly higher methylation levels (*p* < 0.05) of JAM2 in LUAD than normal tissues, which was similar to that in esophageal LUSC and colorectal cancer. We also found that JAM2 was negatively correlated with three DNA methyltransferases DNMT1, DNMT3A and DNMT3B. The downregulation of JAM2 may be due to the hypermethylated status of the JAM2 gene in LUAD.

There is growing evidence showed that the immune system played a role in preventing occurrence, growth, and metastatic diffusion of LUAD [38, 39]. Understanding the role and mechanisms of immune response in LUAD might help to better use immunotherapeutic agents in this disease. Therefore, immune scores were analyzed by the R package ssGSEA, and JAM2 expression was positively associated with antagonizing immune cells, such as NK cells, Th1 cells, iDC, CD8^+^ T cells, and cytotoxic cells. The administration of immune checkpoint modulators have exhibited unexpected antitumor effects in multiple types of cancers. We also estimated the expression of chemokines and found that JAM2 expression was positively related to CCR2, CCL14 and CXCL12. These results suggested that JAM2 plays a broad role in LUAD inflammatory infiltration and higher JAM2 expression may predict a better immunotherapy response. Previous investigation also confirmed that JAM2 expression in stomach adenocarcinoma was significantly correlated with multiple tumor‐infiltrating immune cells [40].

In summary, our research confirmed the aberrant expression of JAM2 in LUAD, which may be the result of methylation. We further validated the prognostic significance of JAM2 and its association with immune infiltration in LUAD. In LUAD cell lines, overexpression of JAM2 could inhibit migratory and invasive capabilities. However, there still need to be more validation experiments to elucidate the underlying mechanism in the future. Taken together, our results showed that JAM2 might be a promising molecular target for early diagnosis and targeted therapy of LUAD.

## Author Contributions

**Jun Chen:** project administration, conceptualization. **Yuan Cui:** project administration, writing – original draft. **Zhimeng Chen:** project administration. **Hao Ding:** software, methodology. **Chang Li:** data curation. **Sheng Ju:** software, methodology. **Cheng Ding:** visualization. **Chun Xu:** resources. **Jun Zhao:** supervision, writing – review and editing. **Xin Tong:** investigation, writing – review and editing.

## Ethics Statement

The authors are accountable for all aspects of the work in ensuring that questions related to the accuracy or integrity of any part of the work are appropriately investigated and resolved. This study was approved by the Ethics Committee of Soochow University.

## Conflicts of Interest

The authors declare no conflicts of interest.

## Supporting information


**Figure S1** The uncropped blotting images of the study.

**Figure S2** Gene set enrichment analysis (GSEA) was applied to explore molecular pathways mediated by JAM2 in LUAD cells.

Table S1.

Table S2.

Table S3.

## Data Availability

The data that support the findings of this study are available from the corresponding author upon reasonable request.
