# Supplementary figures and images for: Aberrant Expression of JAM2 Inhibits Invasion and Migration in Lung Adenocarcinoma
Source: Cancer Rep (Hoboken). 2025 Jan 21;8(1):e70038. doi: 10.1002/cnr2.70038 (PMC11751475; doi:10.1002/cnr2.70038)

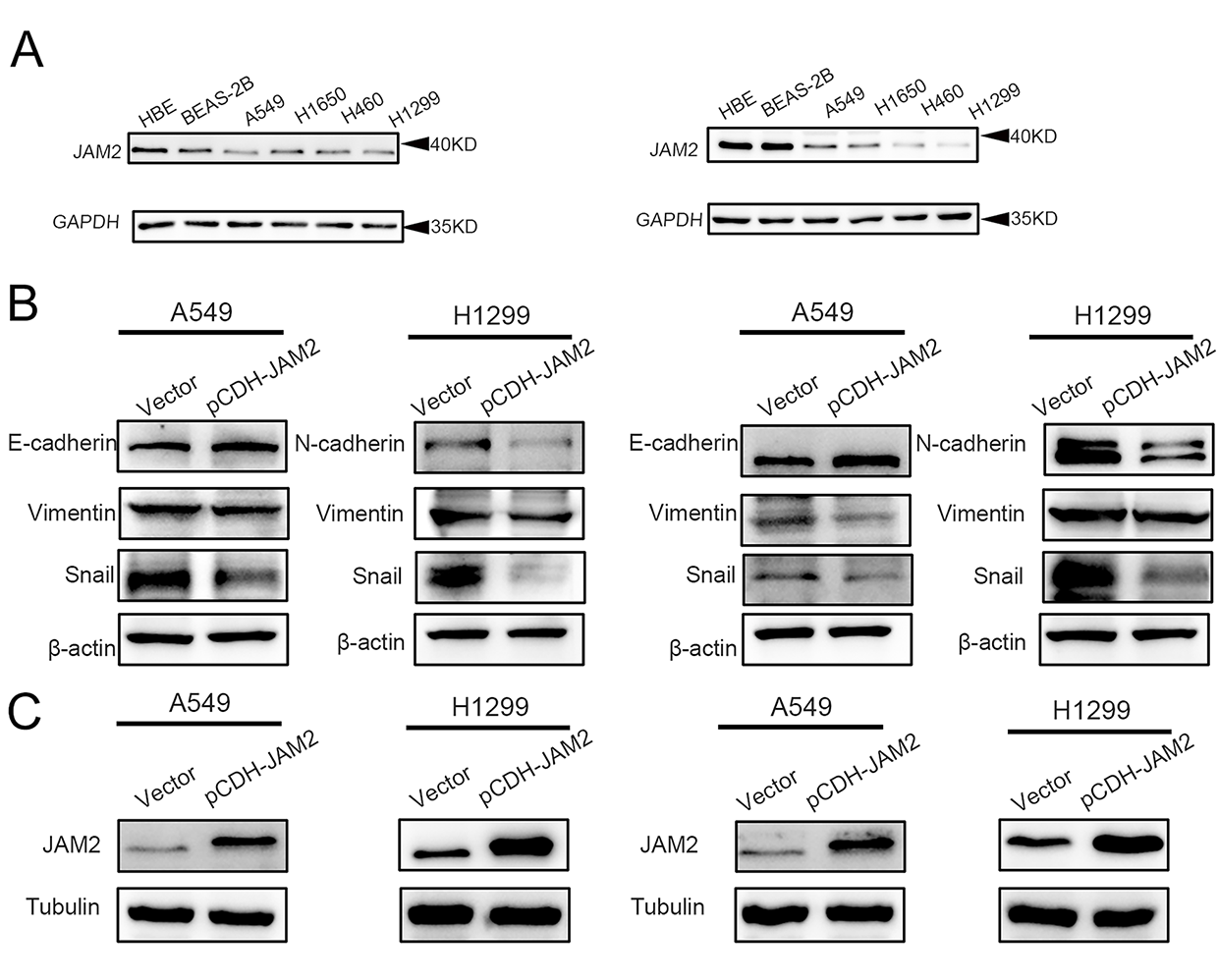

Supplement: Supplementary file 1 — Figure S1 The uncropped blotting images of the study. [file CNR2-8-e70038-s005.tif]

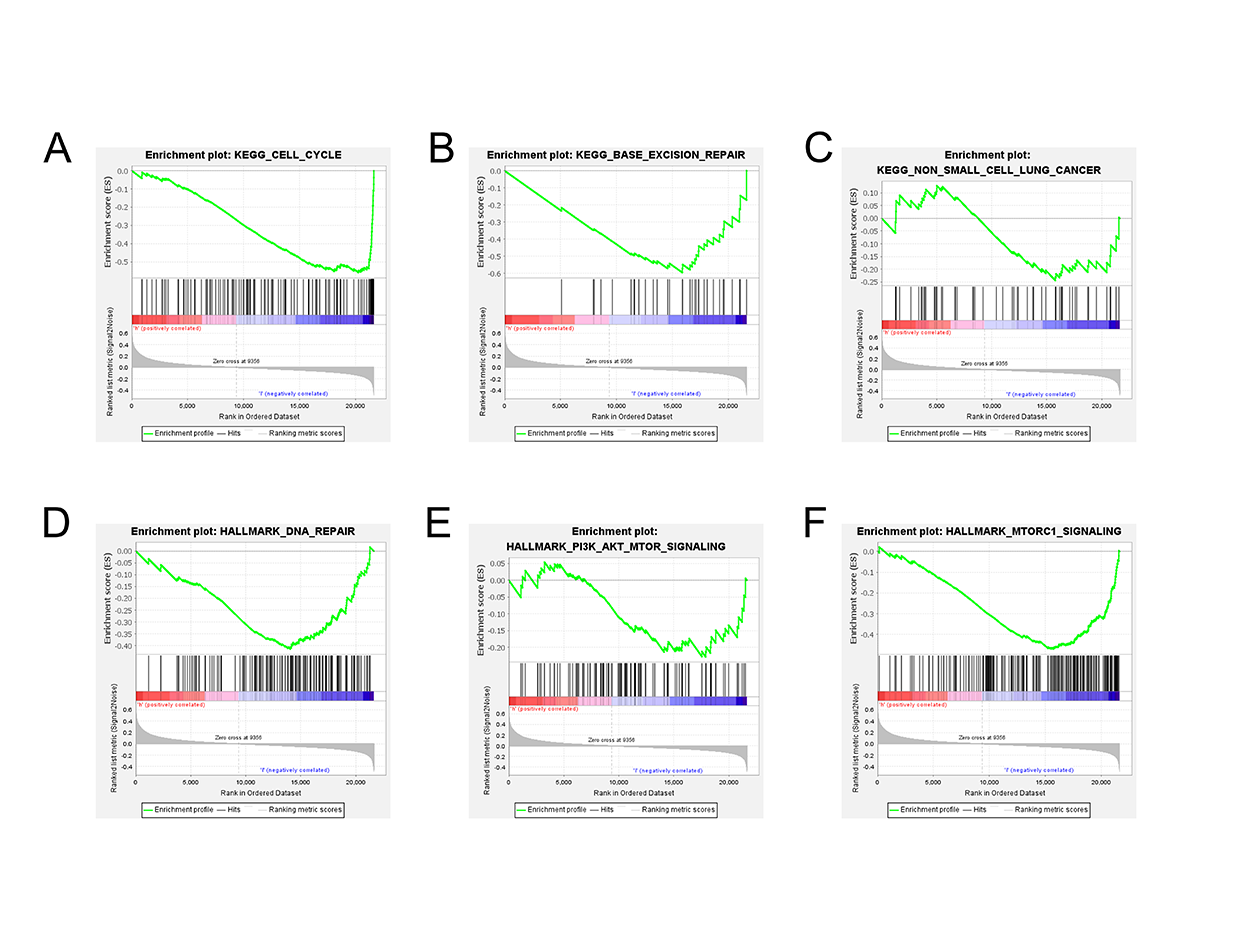

Supplement: Supplementary file 2 — Figure S2 Gene set enrichment analysis (GSEA) was applied to explore molecular pathways mediated by JAM2 in LUAD cells. [file CNR2-8-e70038-s004.tif]
