# Supplementary material for: Aberrant Expression of JAM2 Inhibits Invasion and Migration in Lung Adenocarcinoma
Source: Cancer Rep (Hoboken). 2025 Jan 21;8(1):e70038. doi: 10.1002/cnr2.70038 (PMC11751475; doi:10.1002/cnr2.70038)
Supplement: Supplementary file 5 — Table S3. [file CNR2-8-e70038-s002.pdf]

The table of 215 genes with differential expression value in four datasets

	GSE10072	GSE31210	GSE32863	GSE43458
genes	logFC	logFC	logFC	logFC
ABCA3	-1.045	-1.711	-1.795	-1.521
ABCA8	-1.999	-2.318	-1.256	-2.427
ACADL	-1.738	-2.663	-1.174	-2.609
ADAMTS1	-1.283	-2.245	-2.104	-1.434
ADARB1	-1.889	-1.530	-1.663	-1.141
ADGRL2	-1.923	-1.241	-1.173	-1.909
ADH1B	-1.841	-2.798	-2.664	-2.743
ADIRF	-2.705	-1.958	-2.991	-1.303
AGER	-2.829	-4.794	-3.021	-3.388
AGR2	1.144	1.597	1.260	1.180
AHNAK	-1.352	-1.142	-1.340	-1.058
ANGPT1	-1.060	-1.486	-1.631	-1.582
ANOS1	-2.055	-1.627	-2.417	-2.394
ANXA3	-1.470	-1.505	-1.573	-1.582
AOC3	-1.415	-2.674	-1.099	-1.637
AQP1	-1.263	-1.401	-1.236	-1.495
AQP4	-1.045	-2.019	-1.736	-2.555
ARHGAP44	-1.076	-1.083	-1.587	-1.210
ARRB1	1.007	1.413	1.179	1.140
BCHE	-1.175	-2.516	-1.431	-1.790
C7	-1.178	-1.760	-2.450	-1.625
CA2	1.670	1.199	2.016	1.080
CA4	-1.531	-3.698	-4.011	-1.227
CACNA2D2	-1.364	-1.763	-1.443	-1.923
CALCRL	-1.070	-1.795	-1.954	-2.157
CAT	-1.374	-1.010	-1.572	-1.172
CAV1	-1.585	-1.749	-3.581	-2.313
CAV2	-2.464	-1.778	-2.182	-1.913
CCNB2	1.114	1.280	1.490	1.323
CD24	1.165	1.876	1.035	1.049
CD34	1.050	1.180	1.450	1.036
CD36	-2.567	-2.633	-2.038	-2.243
CD93	-1.180	-1.662	-2.297	-1.428
CDC20	1.029	1.592	2.204	1.095
CDH3	1.329	2.485	2.054	1.755
CDH5	-1.820	-2.241	-2.418	-2.019
CDO1	-1.315	-2.127	-1.357	-1.275
CEACAM5	2.285	2.949	2.556	2.190
CENPF	1.342	1.487	1.232	1.629

**CFB**	1.021	1.434	1.399	1.005
**CLDN18**	-1.454	-3.536	-2.556	-2.428
**CLIC5**	-1.259	-2.606	-2.369	-2.559
**COL10A1**	1.428	4.530	1.383	1.609
**COL11A1**	1.565	4.697	1.851	1.355
**COL1A1**	1.082	2.519	2.674	1.810
**COL3A1**	1.706	1.015	1.534	1.474
**COMP**	1.021	2.854	2.440	1.173
**CPA3**	1.147	1.121	2.722	1.183
**CPB2**	-1.102	-3.327	-1.687	-2.577
**CRABP2**	1.265	2.744	2.835	1.790
**CRTAC1**	-1.206	-1.956	-2.923	-1.327
**CRYAB**	-1.264	-1.136	-2.208	-2.256
**CST1**	1.081	2.949	2.467	2.009
**CTNNAL1**	-1.428	-1.364	-1.333	-1.511
**CXCL2**	-1.119	-1.281	-2.330	-1.506
**CYP4B1**	-2.466	-1.989	-2.703	-2.453
**DACH1**	-1.086	-2.511	-1.624	-1.720
**DCN**	-1.194	-1.035	-2.516	-1.088
**DES**	-1.417	-1.416	-1.215	-1.382
**DPT**	1.131	1.168	1.950	1.351
**DSP**	1.142	1.017	1.665	1.062
**DUOX1**	-2.177	-1.787	-1.060	-1.460
**DUSP1**	-1.075	-1.077	-1.759	-1.058
**ECT2**	1.664	1.050	1.176	1.089
**EDN1**	-1.199	-1.260	-1.079	-1.961
**EDNRB**	-1.700	-2.660	-2.385	-2.713
**EFEMP1**	-1.402	-1.159	-1.393	-1.248
**EMCN**	-1.038	-2.175	-1.695	-2.440
**EMP1**	-1.003	-1.815	-1.299	-1.094
**EPB41L2**	-1.092	-1.002	-1.279	-1.163
**FABP4**	-2.698	-3.619	-3.675	-4.209
**FAM107A**	-1.717	-2.839	-3.877	-1.467
FAM189A2	-1.310	-2.021	-1.844	-2.059
**FBLN5**	-1.106	-1.379	-1.721	-1.320
**FCN3**	-2.877	-2.879	-3.049	-2.804
**FEZ1**	-1.452	-1.538	-2.169	-1.242
**FHL1**	-1.394	-1.751	-2.454	-2.763
**FLRT3**	1.058	1.013	1.421	1.534
**FMO2**	-1.958	-2.043	-3.107	-2.431
**FOSB**	-1.613	-1.659	-3.183	-1.731
**GCNT3**	1.115	4.057	3.217	1.577
**GDF15**	1.662	1.820	1.152	1.249

**GHR**	-1.451	-1.923	-1.371	-1.642
**GNG11**	-1.278	-1.991	-2.634	-2.109
GOLM1	1.831	2.210	1.816	1.500
**GPC3**	-1.943	-1.763	-2.319	-2.198
**GPM6B**	-1.377	-1.894	-1.156	-1.249
**GPRC5A**	1.053	1.212	1.052	1.006
**GPX3**	-1.629	-1.694	-2.105	-1.515
**GRK5**	-1.081	-1.970	-1.411	-1.869
HBA2	-2.652	-2.937	-3.925	-2.438
**HBB**	-2.160	-2.700	-4.143	-2.349
**HBEGF**	-1.424	-1.410	-2.089	-1.550
**HEG1**	-1.269	-1.357	-1.723	-1.641
**HEY1**	-1.269	-1.166	-1.207	-1.318
**HIGD1B**	-1.364	-2.046	-2.521	-1.896
**HMGB3**	1.549	2.069	2.124	1.318
**HOXA5**	1.136	1.650	2.075	1.212
**HSD17B6**	-1.669	-1.067	-2.423	-2.159
**HSPB8**	-1.434	-1.168	-1.823	-1.751
**ICAM2**	-1.002	-1.303	-1.364	-1.207
**ID1**	-1.097	-1.455	-1.289	-1.348
**IGF2BP3**	1.704	1.875	1.216	1.209
**IL1RL1**	-1.557	-1.674	-1.126	-2.171
**IL33**	-1.350	-1.974	-1.055	-1.904
**IL6**	-1.104	-2.720	-2.663	-1.743
**ITM2A**	-1.069	-1.528	-1.385	-1.474
**JAM2**	-1.096	-1.964	-1.879	-1.738
**KDELR3**	1.386	1.677	1.208	1.091
**KIAA0101**	1.685	1.878	1.291	1.039
**KLF4**	-1.178	-1.843	-1.211	-1.878
**KLF6**	-1.030	-1.008	-1.050	-1.029
**LAMP3**	-1.173	-1.881	-2.453	-1.700
**LCN2**	1.367	1.831	1.782	1.296
**LDB2**	-1.773	-1.870	-2.709	-1.818
**LDLR**	-1.389	-1.000	-1.204	-1.101
LGSN	1.366	2.258	1.188	1.209
**LHFP**	1.237	1.359	1.668	1.544
LIMCH1	-1.029	-1.144	-1.567	-1.459
**LMCD1**	-1.172	-1.032	-1.623	-1.056
**LMO7**	-1.056	-1.267	-1.293	-1.060
**LPL**	-1.384	-1.979	-2.143	-1.815
**LRRC32**	1.142	1.471	1.747	1.349
**LRRN3**	-1.292	-2.359	-1.239	-1.926
LYVE1	-1.231	-2.999	-2.689	-2.625

**MARCO**	-1.664	-2.657	-3.018	-1.207
**MELK**	1.047	1.952	1.059	1.146
**METTL7A**	-1.685	-1.206	-1.324	-1.231
**MME**	-1.156	-2.881	-2.273	-2.390
**MMP11**	1.429	2.903	2.990	1.578
**MMP12**	1.568	3.672	1.357	2.585
**MMP9**	1.405	2.343	2.250	1.423
**MSR1**	1.295	1.165	1.055	1.164
**MT1M**	-1.105	-2.956	-2.956	-2.144
**MUC16**	1.394	2.371	1.312	1.348
**MYH10**	-1.110	-1.168	-1.475	-1.283
**MYH11**	-1.658	-1.148	-1.491	-1.282
**MYL9**	-1.057	-1.179	-1.049	-1.085
**NEDD4L**	-1.456	-1.026	-1.010	-1.079
**NQO1**	1.392	1.579	1.694	1.478
**OLFML1**	-1.132	-1.332	-1.581	-1.266
**OLR1**	-1.564	-1.205	-2.399	-1.519
P3H2	-1.397	-1.174	-1.765	-1.542
**PCOLCE2**	-1.548	-2.796	-2.426	-1.184
**PCP4**	1.195	3.073	1.519	1.419
**PDK4**	-1.273	-2.457	-2.205	-2.099
PDZD2	-2.260	-1.862	-1.176	-1.629
**PECAM1**	-1.227	-1.418	-2.260	-1.586
**PGC**	-1.051	-1.908	-3.569	-2.196
**PHACTR2**	-1.005	-1.014	-1.049	-1.060
PID1	-1.536	-1.133	-1.711	-1.036
**PLA2G1B**	-1.027	-1.622	-2.596	-1.670
PLLP	-1.329	-1.094	-1.322	-1.108
PLPP2	1.872	2.205	1.531	1.063
**PTPRB**	-1.071	-2.210	-1.728	-2.356
**PTRF**	-1.237	-1.371	-1.665	-1.309
**RAB11FIP1**	-1.014	-1.002	-1.128	-1.095
**RAMP2**	-1.266	-1.288	-1.437	-1.415
**RAMP3**	-2.266	-2.596	-2.090	-1.156
**RECK**	-1.427	-1.218	-1.132	-1.014
RGCC	-2.243	-1.517	-2.742	-1.628
**S100P**	1.535	1.859	1.766	1.299
S1PR1	-1.277	-2.208	-1.714	-1.599
**SASH1**	-1.114	-1.667	-1.605	-1.609
**SCGB1A1**	-1.658	-2.806	-3.756	-2.560
**SDPR**	-2.259	-2.605	-2.742	-2.272
**SEMA5A**	-1.313	-1.652	-1.264	-1.704
**SEMA6A**	-1.132	-2.132	-2.177	-2.373

**SFN**	1.004	1.860	2.152	1.235
**SFTPC**	-2.319	-3.358	-3.684	-2.834
**SFTPD**	-1.711	-1.648	-2.417	-1.299
**SLC2A1**	1.059	1.421	2.360	1.668
**SLC39A8**	-1.963	-1.050	-1.686	-1.730
**SLC6A4**	-2.090	-4.656	-1.894	-3.985
**SLCO2A1**	-1.516	-1.576	-1.398	-1.493
**SLIT2**	-1.734	-1.760	-1.735	-1.870
**SMAD6**	-1.717	-1.496	-2.193	-1.141
**SOCS2**	-1.163	-1.539	-1.795	-1.263
**SOSTDC1**	-1.718	-5.451	-2.120	-2.937
**SPINK1**	1.991	3.715	3.153	2.582
**SPOCK2**	-2.032	-1.649	-2.925	-1.737
**SPP1**	2.480	3.838	2.947	4.330
**SPTBN1**	-1.279	-1.148	-1.095	-1.248
**SRPX**	-1.377	-1.767	-2.287	-1.377
**STARD13**	-1.220	-1.369	-1.018	-1.334
**STXBP6**	-1.405	-2.990	-1.531	-2.052
**SULF1**	1.198	1.762	1.263	1.447
**SVEP1**	-1.192	-1.624	-2.443	-1.649
**TACC1**	-1.498	-1.116	-1.742	-1.238
**TBX3**	-1.329	-1.757	-1.129	-1.134
**TCF21**	-2.034	-2.575	-2.413	-1.879
**TCN1**	1.015	2.131	1.385	1.165
**TEK**	-1.062	-2.296	-2.639	-2.625
**TFAP2A**	1.106	2.135	1.844	1.316
**TGFBR2**	-1.431	-1.151	-1.181	-1.113
**TGFBR3**	-2.165	-2.122	-2.191	-2.202
**THBS2**	1.685	1.891	1.717	1.588
**TIMP1**	1.295	1.319	1.586	1.139
**TIMP3**	-1.022	-1.122	-2.356	-1.170
**TMEM100**	-2.900	-3.395	-3.023	-3.654
TMEM204	-1.565	-1.395	-1.540	-1.133
**TMEM47**	-1.151	-1.380	-1.638	-1.646
**TMPRSS4**	1.184	5.119	1.199	2.616
**TNFRSF21**	1.334	1.207	1.629	1.151
**TNNC1**	-2.409	-3.651	-2.604	-1.743
**TOP2A**	1.479	2.455	2.515	1.925
TPPP3	-1.668	-1.944	-2.116	-1.330
**TSPAN7**	-1.050	-2.108	-2.179	-1.927
**TYMS**	1.483	1.016	1.030	1.100
**VIPR1**	-2.123	-2.119	-1.988	-1.954
**VSIG4**	-1.584	-1.534	-2.404	-1.254

**VWF**	-1.598	-1.941	-2.228	-1.733
**WASF3**	-1.222	-1.454	-1.163	-1.155
**WIF1**	-2.815	-3.347	-2.253	-3.661
**ZBTB16**	-2.050	-2.527	-1.128	-1.569
